# Integrating machine learning and immune infiltration analysis to identify core genes and construct a diagnostic model for type 2 diabetes mellitus

**DOI:** 10.3389/fendo.2026.1790356

**Published:** 2026-03-10

**Authors:** Fangqin Cui, Li Li, Mingji Hu, Bao Li, Bang Du, Qingqing Fang, Dake Huang, Xiaonan Zhang

**Affiliations:** 1 Department of Pathophysiology, Bengbu Medical University, Bengbu, Anhui, China; 2 Department of Pathology, Anqing 116 Hospital, Anqing, Anhui, China; 3 Department of Orthopedics, The Second People’s Hospital of Bengbu, Bengbu, Anhui, China; 4 The Comprehensive Lab, School of Basic Medical Science, Anhui Medical University, Hefei, Anhui, China; 5 Bengbu Medical University Key Laboratory of Cardiovascular and Cerebrovascular Diseases, Bengbu, Anhui, China

**Keywords:** BLVRB, diagnostic model, machine learning, NCF1, T2DM

## Abstract

**Background:**

Type 2 diabetes mellitus (T2DM) is a prevalent metabolic disorder, and identifying robust biomarkers is crucial for improving diagnosis and understanding its pathogenesis.

**Methods:**

We analyzed the gene expression dataset GSE250283 from the GEO database to identify differentially expressed genes (DEGs). Functional enrichment analyses (GO and KEGG) were performed. A comprehensive evaluation of 113 machine learning algorithm combinations was conducted to select an optimal model for hub gene identification and diagnostic prediction. The expression of key genes was validated using independent datasets and quantitative real-time PCR (qRT-PCR). Immune infiltration analysis, gene regulatory network prediction, and drug interaction analysis were also carried out.

**Results:**

A total of 393 DEGs were identified, primarily enriched in immune-related functions and pathways. The LASSO+GBM hybrid model demonstrated superior relative performance among the tested algorithms and pinpointed six hub genes: LY96, CCR1, BLVRB, TCF3, LILRA2, and NCF1. A logistic regression model based on these genes showed promising predictive accuracy (AUC > 0.75) in both training and testing sets. Validation confirmed that BLVRB and NCF1 were significantly dysregulated. Immune infiltration revealed significant alterations in the immune cell landscape of T2DM patients, with BLVRB and NCF1 showing substantial correlations with various immune cells. Regulatory network analysis suggested hsa-miR-127-5p as a potential upstream regulator of BLVRB, and methylene blue was identified as a potential targeting drug.

**Conclusion:**

This study identifies novel immune-related candidate genes, particularly BLVRB and NCF1, for T2DM. The constructed diagnostic model shows potential for further development and the findings offer new insights into the immune mechanisms and potential therapeutic avenues for T2DM.

## 1 Introduction

Type 2 diabetes mellitus (T2DM) is a prevalent metabolic disorder that frequently leads to severe cardiovascular, renal, and neuropathic complications, yet approximately half of affected individuals remain undiagnosed until such complications emerge (1–3). This diagnostic gap highlights an urgent clinical need for more effective early detection strategies. Although chronic low-grade inflammation is recognized as a key contributor to T2DM pathogenesis, specific immune-related biomarkers suitable for diagnostic application have not been systematically identified (4, 5).

Current T2DM management, while effective in many patients, is constrained by progressive insulin resistance that often requires insulin dose escalation, thereby increasing hypoglycemia and cardiovascular risks (6–9). This therapeutic limitation underscores the need to better understand T2DM pathogenesis and develop novel diagnostic and precision therapeutic strategies.

In the pathophysiology of type 2 diabetes, metabolic abnormalities and chronic inflammation form a self-reinforcing vicious cycle initiated by nutrient excess-induced metabolic stress. Persisten elevation, of circulating free fatty acids and glucose leads to ectopic lipid deposition, endoplasmic reticulum stress, and mitochondrial dysfunction, resulting in excessive reactive oxygen species generation (10–13). These stress signals activate the innate immune system via pattern recognition receptors such as Toll-like receptor 4, triggering IKKβ/NF-κB and JNK inflammatory cascades (14). This process is particularly prominent in expanded adipose tissue, where recruited monocytes differentiate into M1 pro-inflammatory macrophages (15) while lymphocyte subsets including CD4+ Th1 cells synergistically amplify the inflammatory response. The stimulated immune cells release substantial quantities of cytokines, notably tumor necrosis factor-α and interleukin-6, which influence insulin-reactive tissues through paracrine and endocrine mechanisms (16, 17). By inducing serine phosphorylation of insulin receptor substrate-1, these cytokines directly interfere with insulin signal transduction, exacerbating systemic insulin resistance and perpetuating the meta-inflammatory state. This interconnected immune-metabolic dysregulation underscores the need to identify specific immune-related genes that could serve as diagnostic biomarkers, a gap that the present study addresses through systematic identification of differentially expressed genes, development of a robust diagnostic model, and exploration of their immunological roles and therapeutic potential in T2DM.

## 2 Results

### 2.1 Screening for DEGs in type 2 diabetes

**Figure 1:**
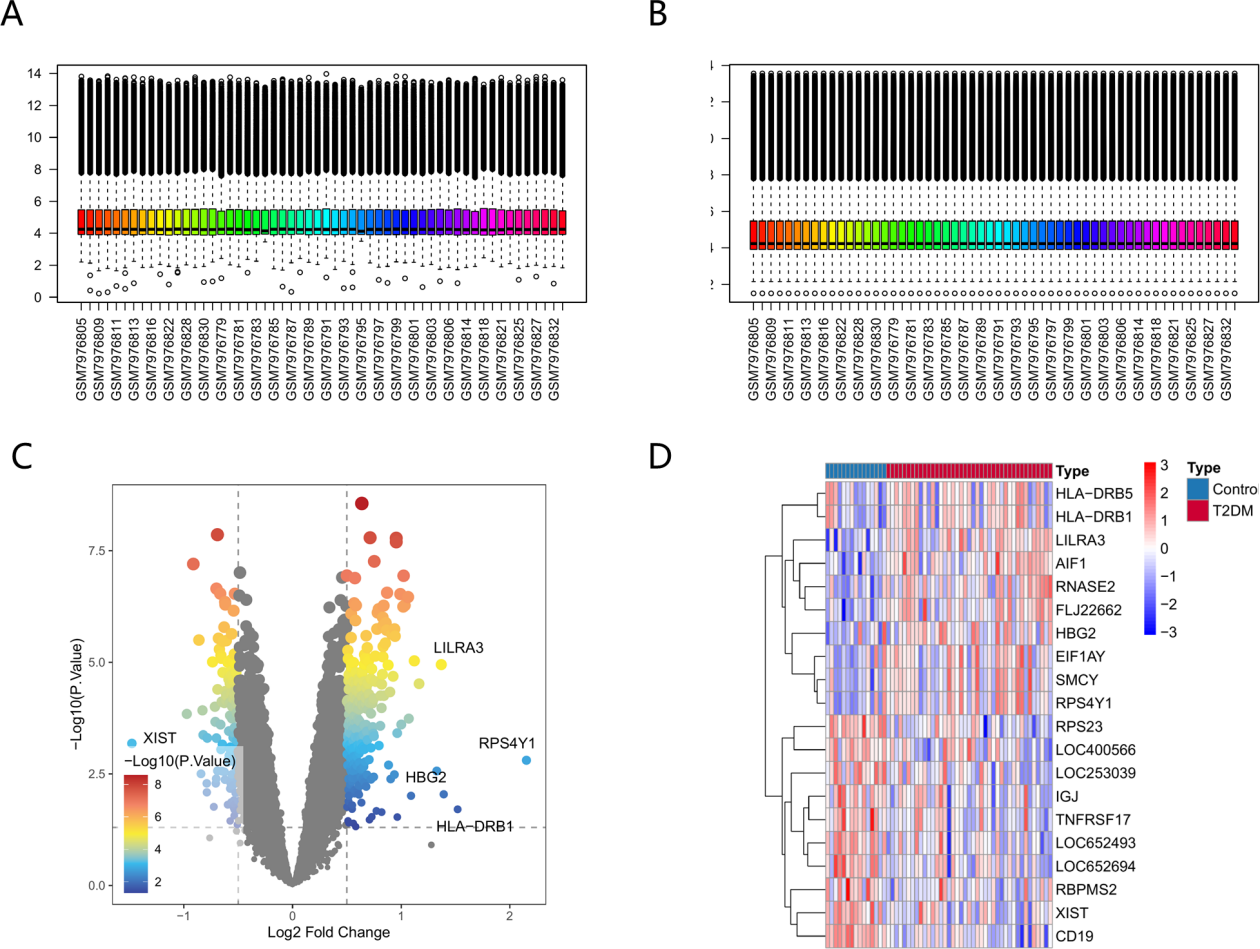
Differential gene expression patterns in type 2 diabetes mellitus. Batch effect correction: A Before **(A)** -and-After **(B)** Comparison. **(C)** The volcano plot illustrating differentially expressed genes in T2DM. **(D)** The heatmap displaying the expression profiles of differentially expressed genes in T2DM.

Type 2 diabetes-associated differentially expressed genes (DEGs) were screened using the GEO dataset GSE250283, comprising 41 patient samples and 15 controls. Following data preprocessing and batch modification (see **Figures 1A, B**), a total of 393 differentially expressed genes (DEGs) were identified, satisfying the conditions of a P-value under 0.05 and a |log2FC| surpassing 0.5. These DEGs’ distribution is illustrated in a volcano plot (**Figure 1C**) and a heatmap (**Figure 1D**).

### 2.2 GO function and KEGG pathway enrichment analysis

**Figure 2:**
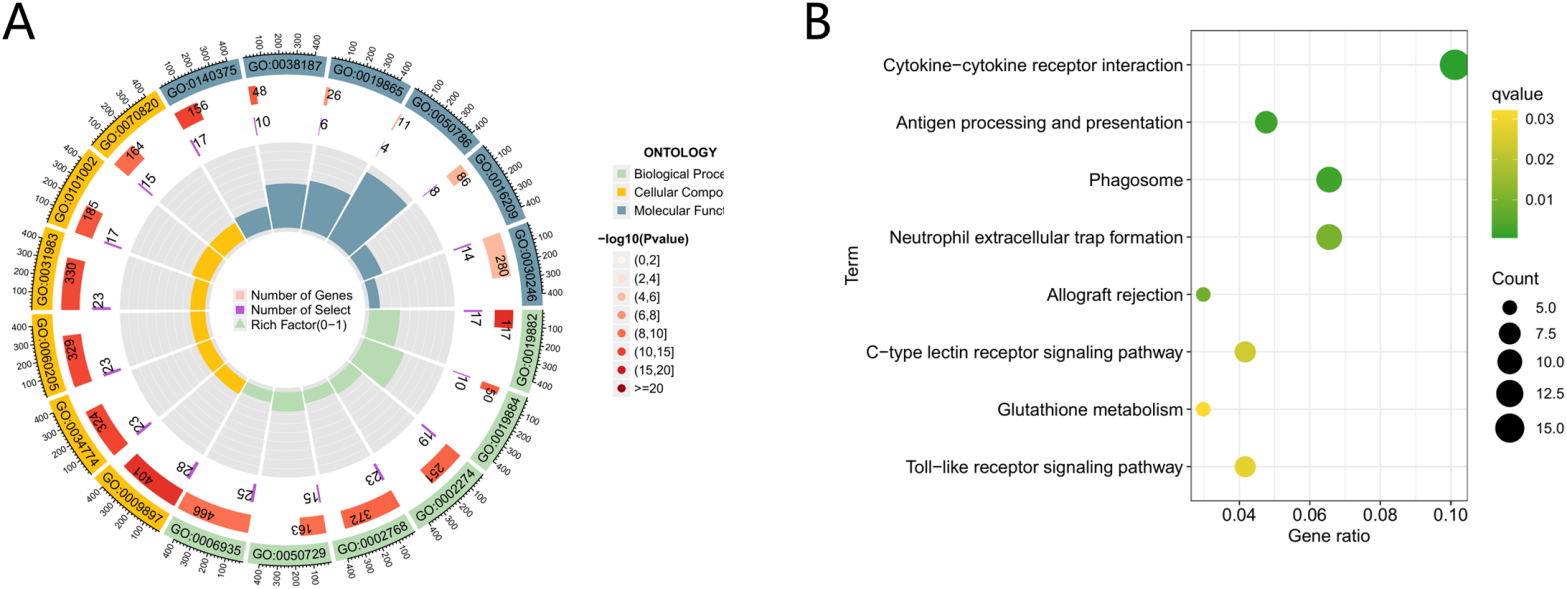
GO and KEGG enrichment analysis. **(A)** Circular plot of GO enrichment analysis. The diagram consists of four concentric rings (from inner to outer): the innermost ring displays GO category labels (Biological Process, Cellular Component, Molecular Function); the second ring shows the number of matched genes (indicated by purple rectangles); the third ring represents the number of enriched genes (shown by pink rectangles, with color intensity reflecting the enrichment level—darker red indicates higher enrichment); and the outermost ring displays the corresponding GO accession numbers for Biological Process terms. **(B)** Clustered dendrogram of KEGG pathway enrichment. In this diagram, the proximity of nodes reflects the degree of similarity between pathways. The vertical axis represents hierarchical relationships, with pathways at lower levels corresponding to broader functional categories. Node color indicates the statistical significance of enrichment, while node size corresponds to the number of genes involved.

To elucidate the biological processes and signaling pathways involved in T2DM-associated genes, enrichment analyses were conducted using Gene Ontology (GO) and Kyoto Encyclopedia of Genes and Genomes (KEGG) resources. The GO analysis identified 166 significantly enriched terms, including 133 biological processes (BP), 23 molecular functions (MF), and 10 cellular components (CC). The KEGG analysis revealed 41 enriched signaling pathways. Among the GO terms, the predominant MF categories were related to immune receptor activity, pattern recognition receptor activity, and immunoglobulin binding. The BP terms were primarily associated with antigen processing and presentation, myeloid leukocyte activation, and regulatory signaling of immune response via cell surface receptors. The CC enrichment was centered in tertiary granules, ficolin-1-rich granules, and secretory granule lumens (**Figure 2A**). Key pathways from KEGG analysis encompassed cytokine-cytokine receptor interaction, C-type lectin receptor signaling, neutrophil extracellular trap formation, and Toll-like receptor signaling (**Figure 2B**).

### 2.3 Leveraging 113 machine learning algorithms for prediction modeling

**Figure 3:**
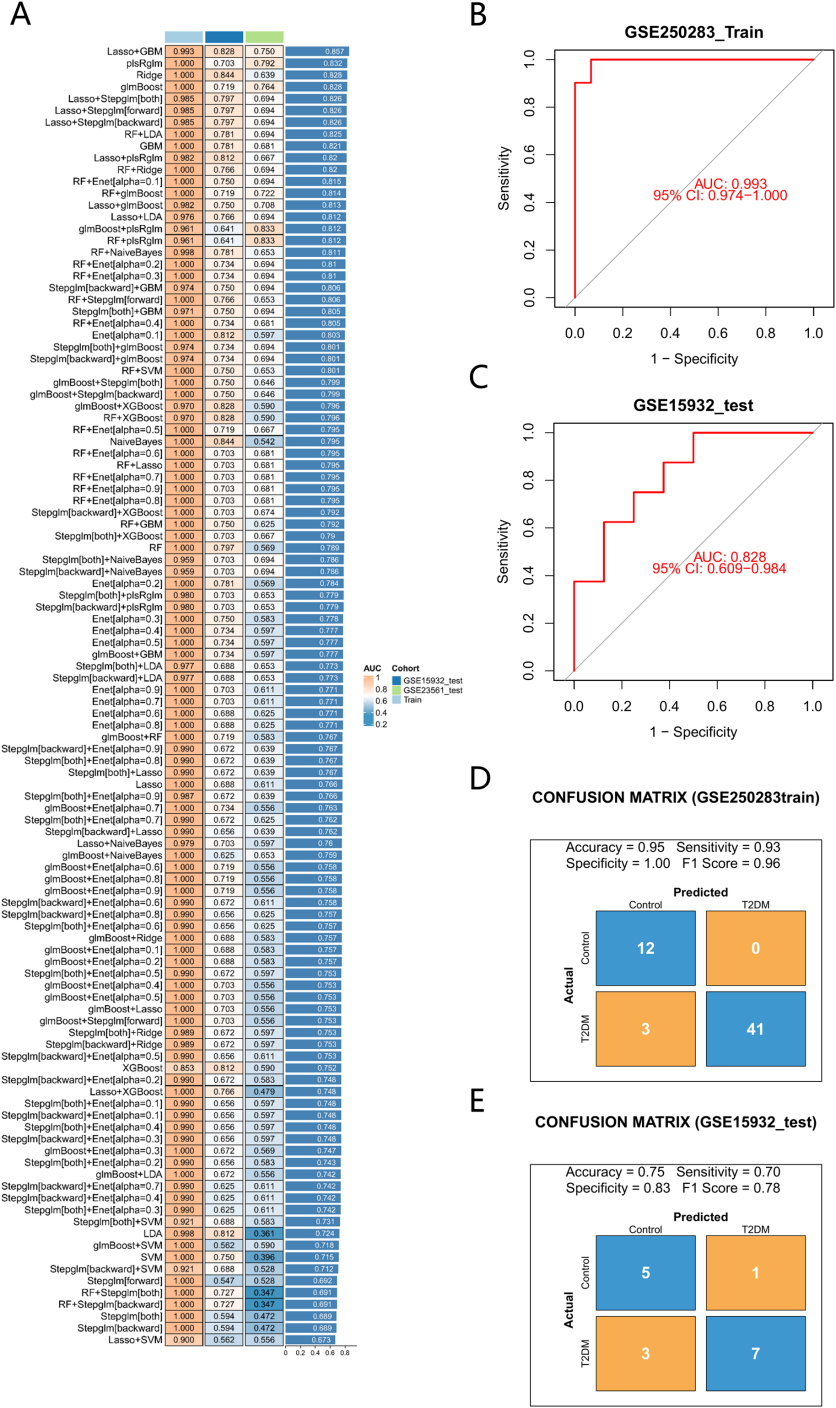
Machine learning prediction model. **(A)** 113 machine learning algorithm combinations. **(B)** AUC values of the model on the training set. **(C)** AUC values of the model on the test set. **(D)** Confusion matrix for the training set. **(E)** Confusion matrix for the test set.

We screened for core genes using 113 distinct combinations derived from 12 fundamental algorithms. Among them, the LASSO+GBM combination achieved the highest mean AUC value when averaged across the training, test, and validation datasets, and was therefore selected as the optimal model. (**Figure 3A**), demonstrating optimal performance, and ultimately identified six core genes: LY96, CCR1, BLVRB, TCF3, LILRA2, and NCF1. To evaluate the performance of the logistic diagnostic model constructed with genes selected by the optimal algorithm, we performed ROC curve analysis and calculated AUC values on both the training set (GSE250283) and the independent test set (GSE15932) (**Figures 3B, C**). The model exhibited encouraging discriminatory ability, evidenced by AUCs surpassing 0.75 in each dataset. Analysis through the confusion matrix revealed the model’s training set accuracy at 0.95, sensitivity at 0.93, specificity at 1.00, and an F1-score of 0.96 (**Figure 3D**). Within the test dataset, the results showed an accuracy rate of 0.75, a sensitivity level of 0.70, a specificity rate of 0.83, and an F1-score of 0.78 (**Figure 3E**). These results collectively indicate that the model holds promise for further validation as a predictive tool.

### 2.4 Evaluation of predictive models

**Figure 4:**
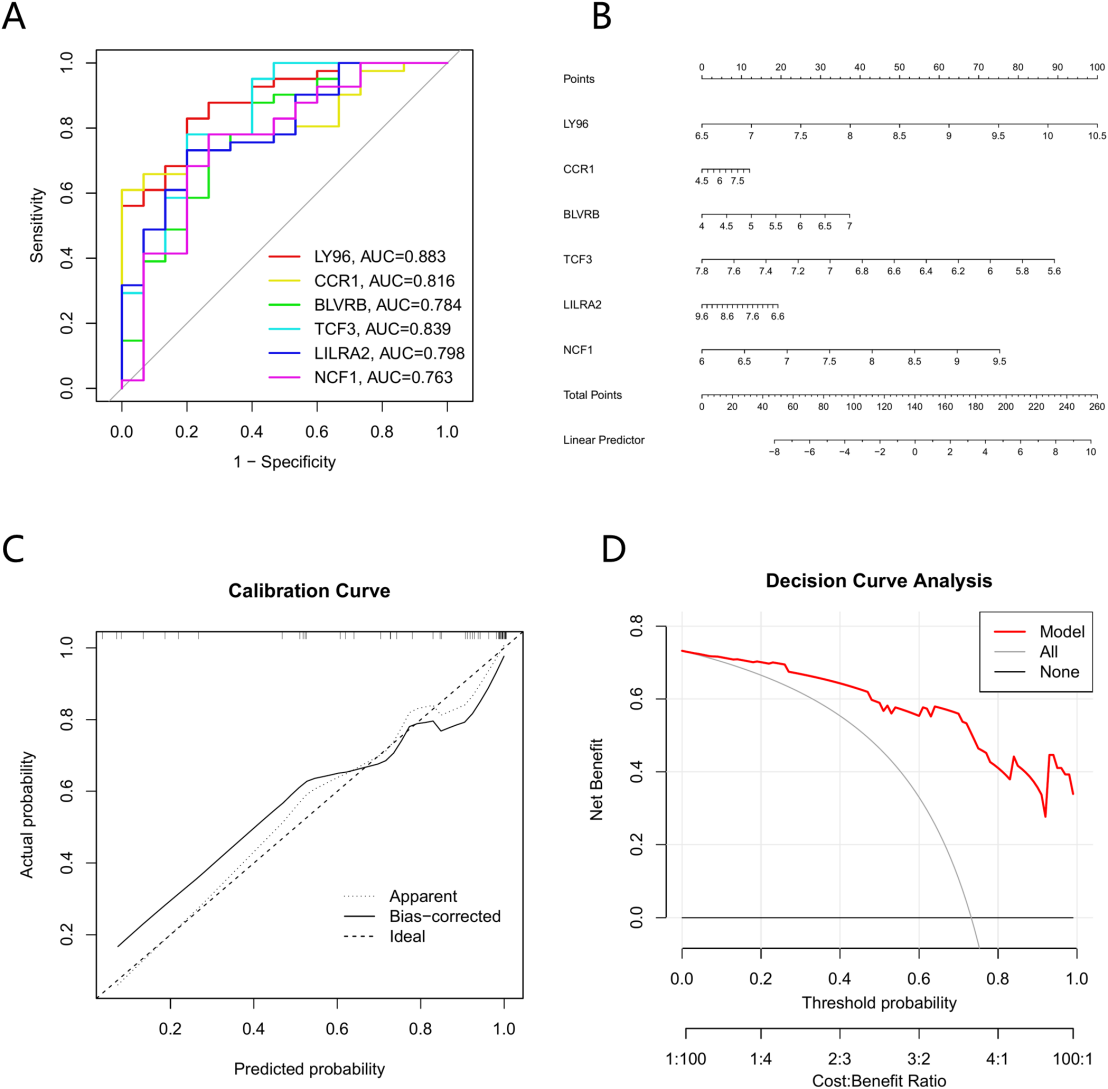
Validation of the 6-gene signature derived from differentially expressed genes. **(A)** ROC curves of the 6-gene signature. **(B)** Diagnostic nomogram incorporating the 6 signature genes. **(C)** Nomogram calibration plot. **(D)** Decision curve analysis (DCA) plot for the nomogram.

To comprehensively evaluate the predictive performance of our model, we calculated the area under the curve (AUC) through receiver operating characteristic (ROC) analysis to assess its ability to discriminate between diseased patients and normal controls (**Figure 4A**). A nomogram was developed to visually demonstrate the interactions among various characteristic genes in the predictive model and their individual contributions to the disease probability (**Figure 4B**). Curves for calibration were created to assess the agreement between forecasted probabilities and the real observed results, confirming the dependability of the model (**Figure 4C**). Additionally, decision curve analysis (DCA) was applied to evaluate the net clinical benefit across various decision thresholds, supporting the model’s practical utility (**Figure 4D**).

### 2.5 Validation of signature gene expression

**Figure 5:**
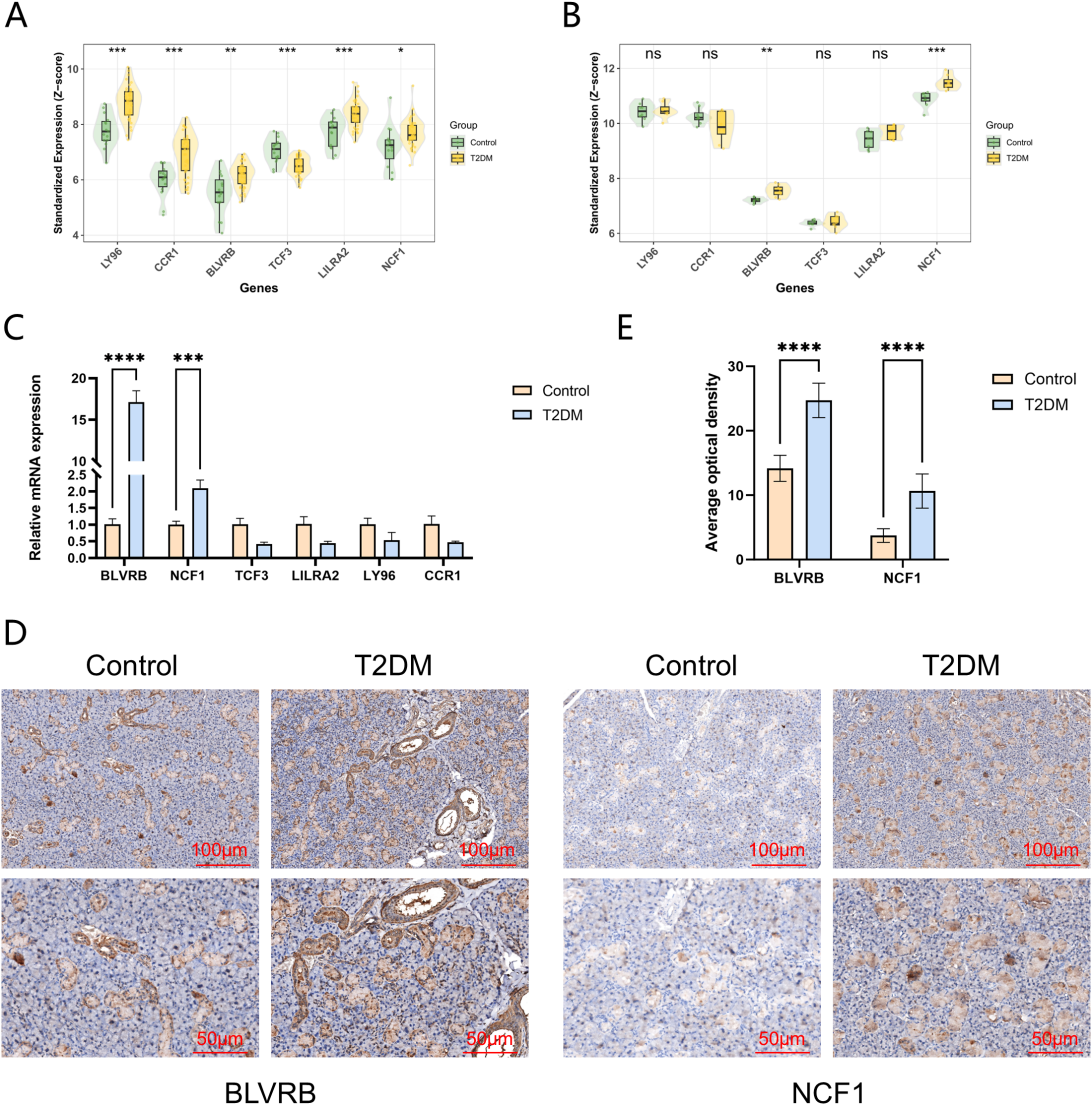
The relative mRNA expression levels of the six key genes. **(A)** Expression profiles of the key genes in the GSE250283 training set. **(B)** Expression profiles of the key genes in the GSE15932 test set. **(C)** qRT-PCR Validation of Key Gene Expression. **(D)** Immunohistochemical stianing of BLVRB and NCF1 in the submandibular glands of rats. **(E)** Quantification of AOD of BLVRB and NCF1 in the submandibular glands of rats. AOD, average optical density. *P < 0.05, **P<0.01, ***P<0.001, ****P<0.0001.

We analyzed the differential expression of six characteristic genes (LY96, CCR1, BLVRB, TCF3, LILRA2, NCF1) in the training set of GSE250283 and the test set of GSE15932. The results showed that in the training set, all these six genes were statistically significant (**Figure 5A**). However, in the test set, only BLVRB and NCF1 still had statistical significance (**Figure 5B**). To further verify these findings, we established a diabetic rat model, extracted peripheral blood from normal rats and diabetic rats for qRT-PCR verification; and extracted submandibular gland tissues from normal rats and diabetic rats for immunohistochemical verification. The qRT-PCR and immunohistochemical results were consistent with the findings of the GSE15932 test set, confirming the differential expression of BLVRB and NCF1 (**Figures 5C–E**).

### 2.6 Immune infiltration analysis in T2DM

**Figure 6:**
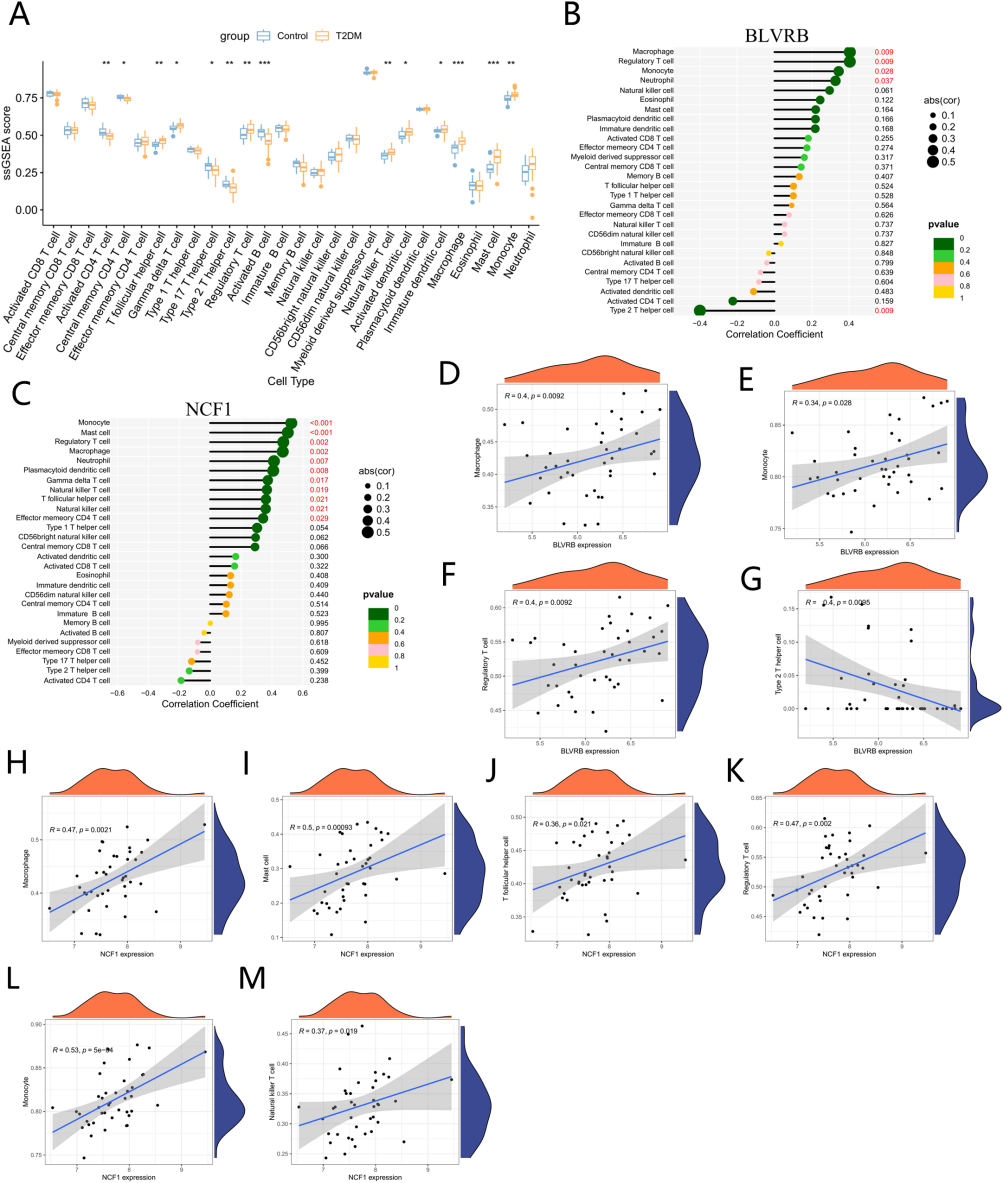
Immune infiltration analysis. **(A)** Immune Cell Proportions in T2DM Patients and Controls. **(B)** Association between BLVRB and Immune Cells in T2DM. **(C)** Association between NCF1 and Immune Cells in T2DM. **(D–G)** Correlation Analyses between Immune Cells and BLVRB. **(H–M)** Correlation Analyses between Immune Cells and NCF1. *P<0.05, **P<0.01, ***P<0.001.

To characterize immune cell infiltration in T2DM, the single-sample Gene Set Enrichment Analysis (ssGSEA) algorithm was applied disease and control groups. Only samples with a ssGSEA deconvolution p-value < 0.05 were retained for analysis. The results revealed significant differences in immune cell composition between the T2DM and control groups. Specifically, the T2DM group demonstrated significantly higher proportions of mast cells, macrophages, monocytes, natural killer cells, T follicular helper cells, and regulatory T cells (Tregs) compared to the control group. Conversely, significantly lower proportions of activated B cells, activated CD4+ T cells, and type 2 T helper cells (Th2) were observed in the T2DM group (**Figure 6A**). Spearman correlation analysis was then conducted between these ssGSEA enrichment scores and the expression levels of BLVRB and NCF1. BLVRB expression correlated positively with macrophages, monocytes, and Tregs, and inversely with Th2 cells (**Figures 6B, D–G**). Similarly, NCF1 showed significant associations with several immune populations, including mast cells, macrophages, monocytes, natural killer cells, T follicular helper cells, and Tregs (**Figures 6C, H–M**). These associations suggest a potential link between these genes and immune cell dynamics in T2DM, warranting further functional investigation.

### 2.7 Molecular regulatory network of BLVRB

**Figure 7:**
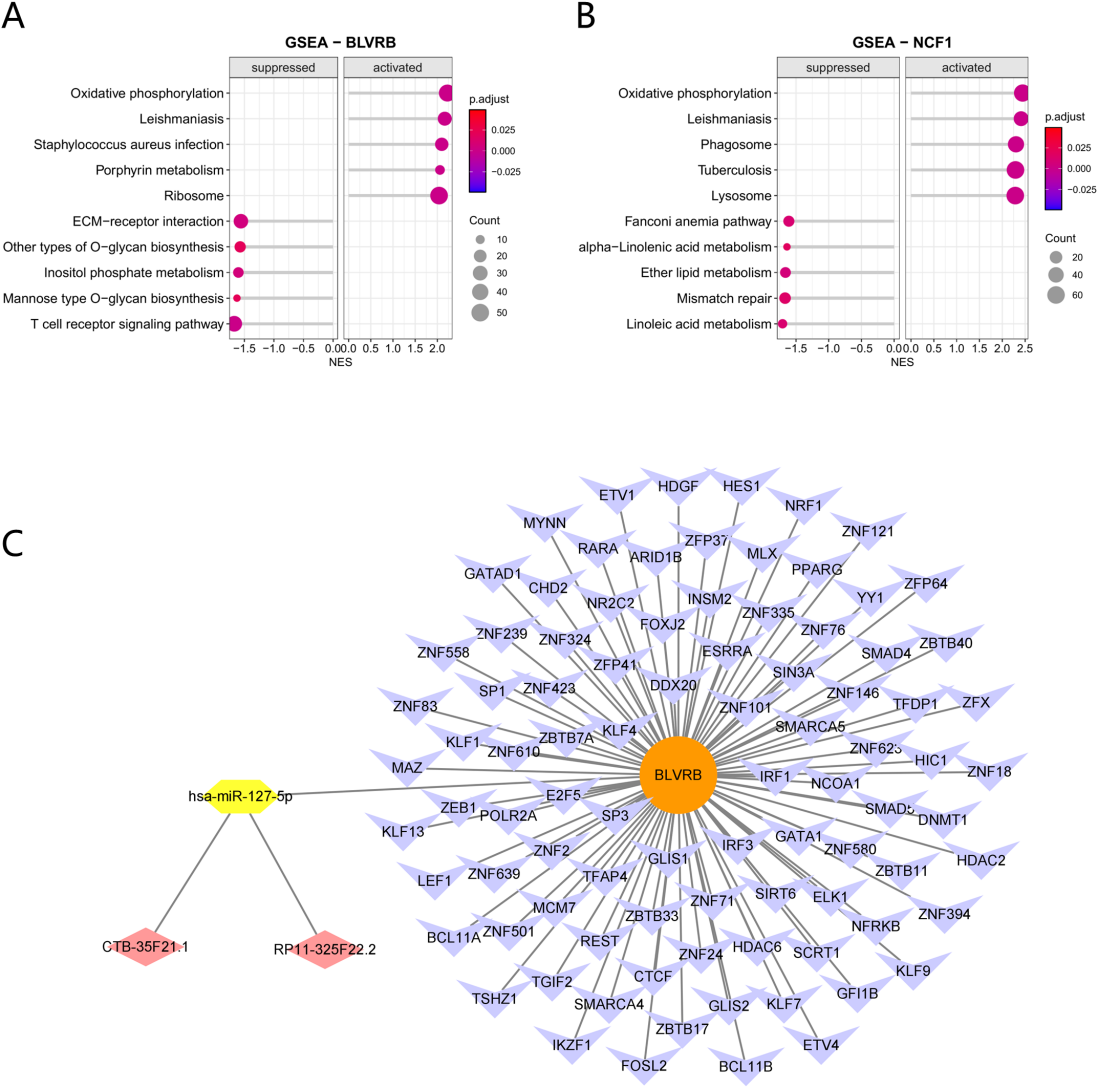
Molecular Regulatory Network. **(A)** GSEA bar plot for BLVRB. **(B)** GSEA bar plot for NCF1. The x−axis represents the Normalized Enrichment Score (NES), indicating the direction and magnitude of enrichment after correction, while the y−axis displays the enriched signaling pathways. Dot size corresponds to the number of enriched genes, and color reflects the adjusted p−value. Bars on the left correspond to suppressed pathways, and bars on the right represent activated pathways. **(C)** lncRNA−miRNA−mRNA−TF regulatory network for BLVRB.

For a deeper understanding of BLVRB and NCF1’s functions in the development of T2DM, Gene Set Enrichment Analysis (GSEA) was conducted to pinpoint the five primary signaling pathways, both increased and decreased, linked to these genes in T2DM. The results demonstrated that both BLVRB and NCF1 were positively correlated with oxidative phosphorylation and leishmaniasis pathways. Notably, BLVRB showed a significant negative correlation with the T cell receptor signaling pathway (**Figures 7A, B**). Additionally, an integrated multi-database approach was employed to establish a comprehensive transcriptional and post-transcriptional regulatory network. Within this network, in silico predictions identified the lncRNAs CTB-35F21.1 and RP11-325F22.2 as potential targets of hsa-miR-127-5p, which in turn is predicted to modulate BLVRB expression, forming a potential ceRNA regulatory axis (**Figure 7C**). This computational model proposes a potential regulatory axis for experimental validation.

### 2.8 Drug interaction analysis of BLVRB

**Figure 8:**
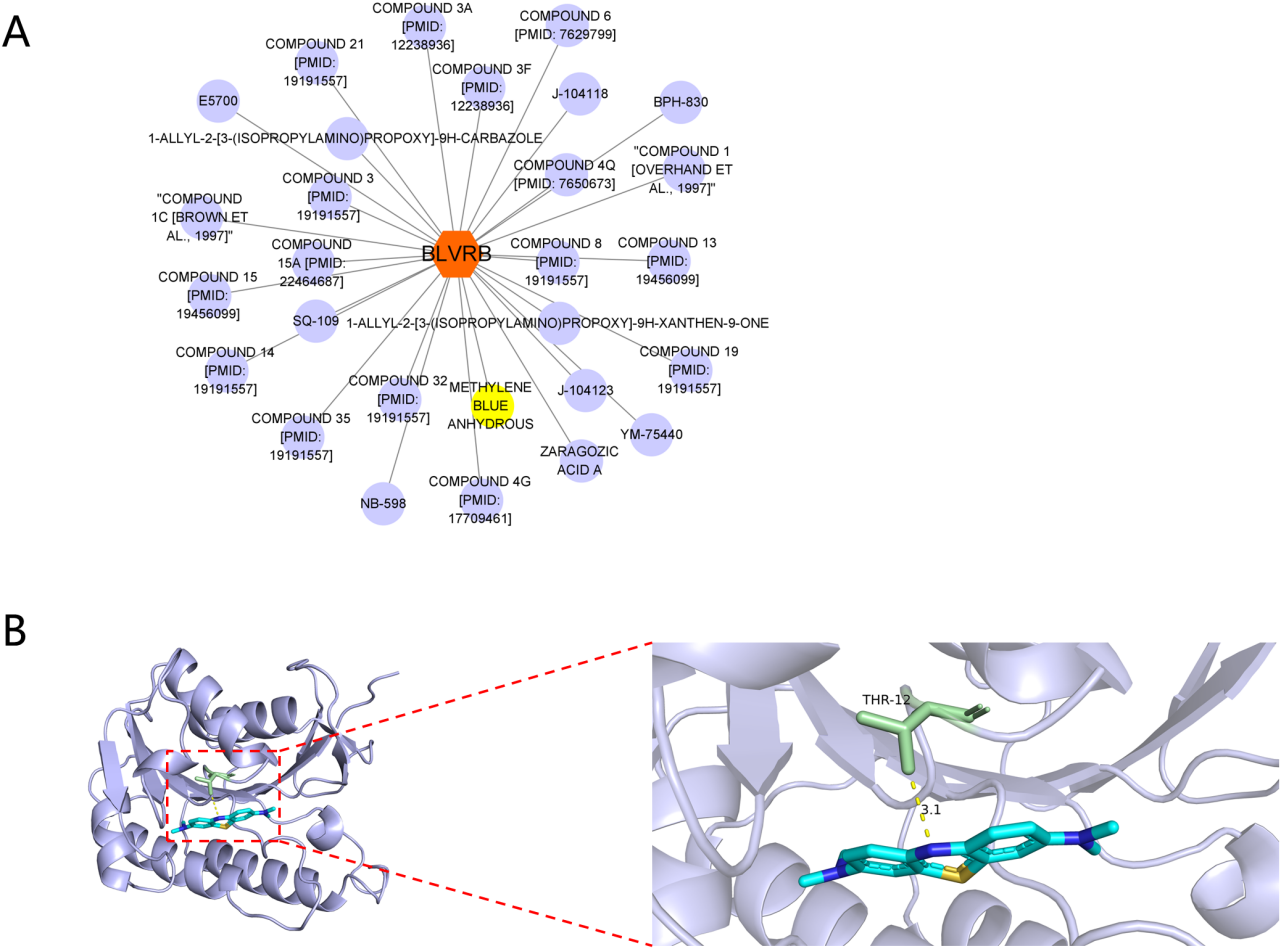
Drug-Gene interactions analysis. **(A)** Drug-BLVRB interaction network. **(B)** Protein-protein docking between BLVRB and targeting drug, methylene blue anhydrous.

Drug-target interaction analysis for the gene BLVRB was performed using the DGIdb database to identify potential therapeutic agents. The results indicated that methylene blue anhydrous was identified as a potential candidate drug (**Figure 8A**). Furthermore, molecular docking was employed to investigate the potential interaction between methylene blue anhydrous and the BLVRB-encoded protein. The binding interaction between BLVRB and methylene blue anhydrous is illustrated in **Figure 8B**.

## 3 Materials and methods

### 3.1 Data preprocessing

Bulk sequencing information for T2DM used in this research was sourced from the Gene Expression Omnibus (GEO) archive (https://www.ncbi.nlm.nih.gov/geo/). The training set GSE250283 comprised 41 T2DM samples and 15 control samples, the validation set GSE15932 included 8 T2DM and 8 control samples, and the test set GSE23561 consisted of 8 T2DM samples and 9 control samples.

### 3.2 Data preprocessing and identification of differentially expressed genes

In this study, the R package GEOquery was used to download relevant datasets from the GEO database, extract expression matrices, and retrieve sample phenotype information. Gene annotation was performed by reading annotation files and matching gene IDs, after which invalid gene IDs were removed and the probe with the highest expression value was retained for each gene. Technical variation between samples was normalized using the TMM method, and raw count data were transformed into log2-counts per million (log2-CPM) values suitable for linear modeling via the voom approach. Inter−sample normalization was further applied using the normalizeBetweenArrays function. Differential expression analysis was carried out using the limma package, with genes defined as differentially expressed (DEGs) at an adjusted P-value < 0.05 and |log2FC| > 0.5 (approximately 1.5-fold change). Results were visualized in volcano plots and heatmaps to display significantly up-regulated, down-regulated, and non-differential genes.

### 3.3 Functional enrichment analysis of DEGs

The ClusterProfiler package in R was utilized to conduct Gene Ontology (GO) and Kyoto Encyclopedia of Genes and Genomes (KEGG) pathway enrichment studies on the differentially expressed genes (DEGs) in T2DM. Results deemed statistically significant were those with a P-value below 0.05.

### 3.4 Machine learning-based discovery of feature genes

A reproducible machine learning pipeline for feature gene discovery was constructed using R software, with GSE250283 designated as the training set and multiple independent cohorts serving as test sets. To ensure rigorous model development, all analytical procedures including differential expression screening, feature selection, and model optimization were strictly confined to the training set. Expression profiles were restricted to genes common across all datasets, with the training set undergoing centering and scaling normalization, while test sets were stratified and normalized by cohort to mitigate batch effects. The pipeline integrated 12 algorithms (Lasso, Ridge, Enet, Stepglm, SVM, LDA, glmBoost, plsRglm, RF, GBM, XGBoost, and NaiveBayes), generating 113 distinct model configurations through combinatorial pairings of feature selection and modeling algorithms. Feature selection was performed on the complete training set to extract genes with non-zero coefficients or importance scores exceeding zero, followed by model training with embedded cross-validation for hyperparameter optimization. Models yielding five or fewer effective features were excluded to ensure robustness, and the remaining models were refitted using logistic regression to enhance interpretability. Model performance was evaluated by calculating AUC values using the “pROC” package, with test sets assessed in a cohort-stratified manner and ranked by mean AUC across multiple cohorts, while comprehensive performance visualization across all models was generated using the “ComplexHeatmap” package.

### 3.5 Immune infiltration analysis

Two complementary methods were used to analyze the immune microenvironment. First, the relative fractions of 22 immune cell types were estimated using the ssGSEA algorithm with the LM22 signature matrix. Sample-level deconvolution results with a p-value ≥ 0.05 were filtered out to ensure reliability. Second, to obtain immune cell enrichment profiles for correlation analysis, ssGSEA was performed using a manually curated gene set representing 28 immune cell types. The ssGSVA package was applied to calculate ssGSEA scores for each immune cell type per sample, reflecting their relative infiltration abundance. For comparative analysis between disease states using ssGSEA-derived fractions, boxplots were generated and statistical significance was assessed using t−tests. For correlation analysis between gene expression and immune infiltration, Spearman correlation coefficients were computed between the ssGSEA enrichment scores and the expression levels of candidate genes, with False Discovery Rate (FDR) correction applied. The correlation results were visualized in a heatmap, where statistically significant associations are denoted by asterisks.

### 3.6 GSEA based on a single gene

To evaluate the links between biomarkers and every gene in the expression matrix, Spearman correlation coefficients were calculated, leading to the creation of a gene-correlation matrix for each biomarker. These coefficients were also used to determine the connections among biomarkers and other genes in the matrix, culminating in a gene-correlation matrix for each biomarker. Based on this correlation matrix, KEGG pathway enrichment analysis was performed for each biomarker using Gene Set Enrichment Analysis (GSEA). The enrichment analysis was implemented with the clusterProfiler package, with a significance threshold set at an adjusted P−value < 0.05. Finally, the KEGG enrichment results for each biomarker were visualized by displaying the top five entries ranked by the absolute value of the Normalized Enrichment Score (NES).

### 3.7 Molecular regulatory network of BLVRB

The molecular regulatory network centered on BLVRB was constructed through an integrated approach. Transcription factors (TFs) interacting with the biomarkers were identified using NetworkAnalyst (https://www.networkanalyst.ca/) and a corresponding biomarker-TF network was visualized with Cytoscape. Potential miRNA regulators were predicted by cross-referencing the database: miRTarBase (https://awi.cuhk.edu.cn/~miRTarBase/miRTarBase_2025/php/index.php). High-confidence miRNA-target interactions were selected based on multi-database support. lncRNA-miRNA interaction data were then obtained from spongeScan (http://mirtoolsgallery.tech/mirtoolsgallery/node/1798). By consolidating TF, miRNA, and lncRNA interactions, a comprehensive lncRNA-miRNA-mRNA-TF regulatory network was established.

### 3.8 Drug prediction

Based on the identified biomarkers, drug−target interaction analysis was performed for these genes using the DGIdb database (https://dgidb.org/). A drug−biomarker interaction network was subsequently visualized with Cytoscape. By mapping the interactions between drugs and their target genes, this network enables the prediction of candidate drugs that may act on these biomarkers. To further explore potential drug molecules that could target the proteins encoded by these biomarkers, molecular docking analysis was conducted. First, the protein identifiers corresponding to the biomarkers were retrieved from the UniProt database (https://www.uniprot.org/), and three−dimensional structural data (in PDB format) were downloaded from the Protein Data Bank (PDB) (https://www.rcsb.org/). The molecular structures (in SDF format) of the predicted candidate drugs were obtained from the PubChem database (https://pubchem.ncbi.nlm.nih.gov/). Next, molecular docking between the key proteins and compounds was carried out using CB−Dock2 (http://183.56.231.194:8001/cb-dock2/index.php) to calculate binding free energy and evaluate intermolecular binding stability. Finally, the docking results were visualized with PyMOL (version 2.5.4) to intuitively analyze the binding sites between the compounds and target proteins.

### 3.9 Construction of diabetic animal models

This study was conducted in accordance with protocols approved by the Institutional Animal Care and Use Committee at Bengbu Medical University (Ethics Approval No. 2024-470). Twenty male SD rats weighing 110g - 160g, sourced from Jiangsu Qinglongshan Biotechnology Co., Ltd. (Animal Production Lincense: SCXK 2024-0001), were randomly allocated into two groups: a control group (n=10) and a diabetic model group (n=10). After a 12-hour fast, baseline fasting blood glucose (FBG) levels were measured. The control group received a standard diet. To induce a type 2 diabetes (T2DM) phenotype, rats in the diabetic group were first fed a high-fat diet (HFD; composition: 2% cholesterol, 10% lard, 88% basal feed) for 8 weeks to induce insulin resistance, followed by a single intraperitoneal injection of streptozotocin (STZ, 2% in 0.1 M citrate buffer, pH 4.5) at a dose of 35 mg/kg. One week post-STZ injection, FBG was measured again, and rats with FBG levels sustained above 7.0 mmol/L were confirmed as diabetic. All animals were then maintained on a standard diet for an additional 8-week experimental period. Body weight and FBG were monitored at biweekly intervals. At the study endpoint, serum was collected for fasting insulin quantification using a commercial ELISA kit (Mercodia), and the Homeostatic Model Assessment of Insulin Resistance (HOMA-IR) was calculated as [FBG (mmol/L) × Fasting Insulin (mIU/L)]/22.5. The sample size provided >80% statistical power to detect significant differences based on preliminary data, and investigators were blinded to group allocation during all outcome assessments.

### 3.10 Quantitative real-time PCR

Total RNA was isolated from peripheral blood of control and diabetic rats using TRIzol reagent. Reverse transcription to cDNA was performed with HiScript II Q RT supermix (Vazyme, Nanjing, China). Quantitative real-time PCR (qRT-PCR) was then carried out using ChamQ universal SYBR qPCR master mix, with β-actin serving as the reference gene for normalization. Each sample was run in triplicate, and relative gene expression levels were calculated via the 2^−ΔΔCt method. Statistical comparisons between groups were performed using an unpaired Student’s t-test, with a p-value < 0.05 considered significant.

### 3.11 Immunohistochemistry

Rat submandibular gland tissues were fixed in 4% paraformaldehyde, dehydrated, cleared, embedded in paraffin, and sectioned. Sections were deparaffinized, rehydrated, subjected to antigen retrieval, and blocked with serum. Primary antibody incubation was performed overnight at 4°C with specific primary antibodies: anti-BLVRB (1:200, Santa cruz, sc-373692) and anti-NCF1 (1:200, Santa cruz, sc-17844). Color development was carried out with DAB solution, and nuclei were counterstained. After dehydration and mounting, slides were examined microscopically. Image analysis was conducted using ImageJ software, with average optical density (AOD) calculated as integral optical density (IOD) divided by positively stained area.

### 3.12 Statistical analysis

Statistical analyses were conducted with R software and GraphPad Prism (version 8.0.2). Group comparisons between two conditions utilized the t-test, whereas comparisons across multiple groups employed one-way analysis of variance (ANOVA). A p-value < 0.05 was defined as statistically significant.

## 4 Discussion

While chronic low-grade inflammation is recognized as a key pathophysiological component of T2DM, systematic identification of specific immune-related genes suitable for diagnostic application has remained limited (18, 19). In this study, we addressed this gap by integrating machine learning and immune infiltration analysis to identify and validate BLVRB and NCF1 as candidate biomarkers, and to construct a diagnostic model with favorable predictive performance across independent cohorts.

Our enrichment analysis revealed that differentially expressed genes in T2DM were significantly concentrated in immune-related pathways, particularly pattern recognition receptor activity, antigen processing, and signaling cascades including Toll-like receptor and neutrophil extracellular trap formation pathways. These findings provide direct transcriptional evidence linking specific immune gene signatures to the metaflammatory state in T2DM, consistent with previous mechanistic studies (20–22), and support our selection of immune-related genes for subsequent diagnostic modeling.

Through systematic integration of 113 machine learning algorithm combinations, we identified LASSO+GBM as the optimal approach, yielding six core genes with promising diagnostic performance. Notably, only BLVRB and NCF1 maintained significant differential expression across both training and independent validation cohorts, a finding further corroborated by qRT−PCR and immunohistochemistry (IHC) in an independent rat model. This multi-platform validation across datasets and experimental systems strongly suggests that BLVRB and NCF1 represent stable, non-dataset-specific candidate biomarkers with potential for clinical translation.

Our immune infiltration analysis revealed significant compositional shifts in T2DM, characterized by elevated mast cells, macrophages, monocytes, natural killer (NK) cells, and regulatory T cells (Tregs). These findings align with established pathological features including adipose tissue macrophage accumulation and impaired immune tolerance (23), and provide a specific immune landscape context for interpreting the potential functional roles of our identified biomarkers BLVRB and NCF1.

Correlation analysis further delineated distinct immune association patterns for our two validated biomarkers: BLVRB positively correlated with macrophages, monocytes, and Tregs while negatively correlating with Th2 cells, suggesting its potential involvement in macrophage polarization and T-cell subset regulation. NCF1, a NADPH oxidase component critical for oxidative burst (24, 25), demonstrated significant associations with mast cells, NK cells, and Tfh cells. These immune correlation profiles, together with their consistent differential expression across cohorts, position BLVRB and NCF1 as plausible molecular links within the inflammation-oxidative stress axis and suggest their potential as therapeutic targets for inflammatory subtypes of T2DM (26).

Single−gene GSEA further revealed that high BLVRB expression was significantly negatively correlated with T−cell receptor signaling pathway activity. This pathway-level finding provides a functional mechanism linking BLVRB to the immune infiltration phenotypes we observed, particularly elevated Tregs, and suggests that BLVRB may contributes to T2DM-associated immune dysregulation at least in part through modulating T−cell activation.

To explore potential upstream regulatory mechanisms, we constructed a computationally predicted BLVRB−related competing endogenous RNA (ceRNA) network. Analysis identified the CTB−35F21.1/RP11−325F22.2-hsa−miR−127−5p – BLVRB axis as a potential key post−transcriptional regulatory pathway, While entirely based on in silico predictions, this finding provides a novel and testable hypothesis for future research on RNA−level regulation in T2DM, which requires experimental validation.

Drug−gene interaction database analysis coupled with molecular identified methylene blue anhydrous as a potential BLVRB-targeting compound with favorable binding affinity. Given its established antioxidant and mitochondrial-enhancing properties, we propose that methylene blue may exert immunometabolic effects in T2DM through BLVRB modulation, representing a candidate drug-repurposing strategy. This computational prediction provides a testable hypothesis for subsequent experimental validation and translational investigation.

Several limitations of this study should be acknowledged. First, our analyses were conducted using peripheral blood datasets, which may not fully capture immune alterations within key metabolic tissues such as pancreas and adipose tissue. Second, the observed correlations between BLVRB, NCF1, and immune subsets are associative and require functional validation through gain- and loss-of-function studies. Finally, the predicted BLVRB-methylene blue interaction is computationally derived and necessitates experimental confirmation via binding assays and preclinical efficacy studies.

## 5 Conclusion

This study identified six core genes through a multi−algorithm integration strategy and established a logistic regression model that demonstrated encouraging discriminative ability in both training and test sets, suggesting its potential as a starting point for the development of an early diagnosis tool for T2DM. In patients with T2DM, there was a notable increase in BLVRB and NCF1 levels, which were linked to the penetration of various immune cell types, indicating their possible involvement in immune control throughout the advancement of T2DM. The expression of BLVRB may potentially be modulated by a computationally predicted lncRNA−miRNA regulatory axis, such as CTB−35F21.1/hsa−miR−127−5p, which warrants further experimental investigation. Drug−target prediction indicated that methylene blue could serve as a potential inhibitor of BLVRB, providing a novel direction for immunotargeted therapy in T2DM. Overall, this work highlights the important role of immune−related genes in the pathogenesis of T2DM and lays a foundation for subsequent functional experiments and clinical translational research. Subsequent investigations should verify the functions of BLVRB and NCF1 via *in vitro* and *in vivo* approaches and evaluate their clinical potential as biomarkers or therapeutic targets.

## Data Availability

Publicly available datasets were analyzed in this study. This data can be found here: All data used in this work can be acquired from the Gene-Expression Omnibus (GEO; https://www.ncbi.nlm.nih.gov/geo/) under the accession number GSE250283, GSE15932 and GSE23561, and the GDC portal (https://portal.gdc.cancer.gov/).
